# Decomposition Analysis of Depressive Symptom Differences Among Older Adults With Hypertension Between Urban and Rural Areas: Cross-Sectional Study

**DOI:** 10.2196/52536

**Published:** 2024-08-01

**Authors:** Lei Yuan, Qinqin Jiang, Yuqing Liu, Yijun Liu, Maolin Du, Jinhai Sun, Meina Li

**Affiliations:** 1 Department of Health Management, Faculty of Military Health Service, Naval Medical University, No. 800 Xiangyin Road, Shanghai, 200433, China, 86 021 81871450; 2 Department of Emergency, Naval Medical Center, Naval Medical University, Shanghai, China; 3 Department of Office, Naval Medical University, Shanghai, China; 4 Department of Military Health Service, Faculty of Military Health Service, Naval Medical University, Shanghai, China

**Keywords:** depression, older, hypertension, Fairlie decomposition, China, older adult, elderly

## Abstract

**Background:**

Hypertension is the most prevalent chronic disease among China’s older population, which comprises a growing proportion of the overall demographic. Older individuals with chronic diseases have a higher risk of developing depressive symptoms than their healthy counterparts, as evidenced in China’s older population, where patients with hypertension exhibit varying rates of depression depending on residing in urban or rural areas.

**Objective:**

This study aimed to investigate factors influencing and contributing to the disparities in depressive symptoms among older urban and rural patients with hypertension in China.

**Methods:**

We used a cross-sectional study design and derived data from the 8th Chinese Longitudinal Health Longevity Survey of 2018. The Fairlie model was applied to analyze the factors contributing to disparities in depressive symptoms between urban and rural older populations with hypertension.

**Results:**

The sample size for this study was 5210, and 12.8% (n=669) of participants exhibited depressive symptoms. The proportions of depressive symptoms in rural and urban areas were 14.1% (n=468) and 10.7% (n=201), respectively. In rural areas, years of education (1-6 years: odds ratio [OR] 0.68, 95% CI 1.10-1.21; ≥7 years: OR 0.47, 95% CI 0.24-0.94), alcohol consumption (yes: OR 0.52, 95% CI 0.29-0.93), exercise (yes: OR 0.78, 95% CI 0.56-1.08), and sleep duration (6.0-7.9 hours: OR 0.29, 95% CI 0.17-0.52; 8.0-9.9 hours: OR 0.24, 95% CI 0.13-0.43; ≥10.0 hours: OR 0.22, 95% CI 0.11-0.41) were protective factors against depressive symptoms in older adults with hypertension, while gender (female: OR 1.94, 95% CI 1.33-2.81), self-reported income status (poor: OR 3.07, 95% CI 2.16-4.37), and activities of daily living (ADL) dysfunction (mild: OR 1.69, 95% CI 1.11-2.58; severe: OR 3.03, 95% CI 1.46-6.32) were risk factors. In urban areas, age (90-99 years: OR 0.37, 95% CI 0.16-0.81; ≥100 years: OR 0.19, 95% CI 0.06-0.66), exercise (yes: OR 0.33, 95% CI 0.22-0.51), and sleep duration (6.0-7.9 hours: OR 0.27, 95% CI 0.10-0.71; 8.0-9.9 hours: OR 0.16, 95% CI 0.06-0.44; ≥10.0 hours: OR 0.18, 95% CI 0.06-0.57) were protective factors, while years of education (1-6 years: OR 1.91, 95% CI 1.05-3.49), self-reported income status (poor: OR 2.94, 95% CI 1.43-6.08), and ADL dysfunction (mild: OR 2.38, 95% CI 1.39-4.06; severe: OR 3.26, 95% CI 1.21-8.76) were risk factors. The Fairlie model revealed that 91.61% of differences in depressive symptoms could be explained by covariates, including years of education (contribution 63.1%), self-reported income status (contribution 13.2%), exercise (contribution 45.7%), sleep duration (contribution 20.8%), ADL dysfunction (contribution −9.6%), and comorbidities (contribution −22.9%).

**Conclusions:**

Older patients with hypertension in rural areas had more depressive symptoms than their counterparts residing in urban areas, which could be explained by years of education, self-reported income status, exercise, sleep duration, ADL dysfunction, and comorbidities. Factors influencing depressive symptoms had similarities regarding exercise, sleep duration, self-reported income status, and ADL dysfunction as well as differences regarding age, gender, years of education, and alcohol consumption.

## Introduction

Hypertension is the most prevalent chronic disease among the older population in China [1-3]. Results from a study of 1,738,886 Chinese participants with an average age of 55.6 years revealed that 44.7% of the participants had hypertension [4], highlighting the urgent need to focus on hypertension as a growing chronic disease in Chinese public health. China is currently facing the challenge of an aging population. According to the 2022 China Statistical Yearbook [5], the proportion of the population aged >60 years was 18.94%, which is more than 200 million people. A forecast study estimated that the proportion of people aged 65 years and older in the total population is expected to increase to approximately 31.71% by 2050 [6]. This significant aging population trend in China also suggests that the number of Chinese people with hypertension may have increased in recent years [7].

Many studies have demonstrated that patients with hypertension are at a higher risk of developing depression [8-11]. Unfortunately, the prevalence of depression is increasing rapidly worldwide, and disability-adjusted life years due to depression rose by 61.1% in 2019 compared to 1990, making it one of the leading disabling factors for adults globally [12]. China is also experiencing an increase in the number of people with depressive symptoms, which is compounded by a shortage of psychiatric medical resources, resulting in the medical needs of 90.5% of the Chinese population with depression not being met [13].

Moreover, various studies have highlighted the mental health disparities between rural and urban areas in China [14-17]. Urban populations enjoy better access to quality health care, education, and a healthier environment, resulting in better physical and mental health and a longer life expectancy compared to their rural counterparts. With the increase in China’s aging population and the widening urban-rural divide, coupled with a shortage of mental health resources, the prevalence of depressive symptoms among urban and rural residents with hypertension is becoming increasingly unequal, posing a major challenge to mental health research in China [16,18].

As the older population in China grows, the demand for health care resources also increases [19]. This group requires the most high-quality health services for prevention, promotion, treatment, rehabilitation, education, palliative care, and end-of-life care [20]. However, the development of geriatric and rehabilitation medicine in China has yet to fully meet the health needs of the older population and faces challenges, such as a shortage of medical resources, overcrowding, and overburdened operations [21]. The question of how China can achieve healthy aging and longevity extends to the field of mental health.

Due to the cumulative effect of health, patients with chronic diseases are more likely to develop depressive symptoms than healthy older individuals [22,23]. A phenomenon has been confirmed among older individuals in China, where patients with hypertension in urban and rural areas exhibit different rates of depression [15,17]. These two populations have shown different distributions in social and demographic characteristics, health status, and lifestyle factors. Although some studies have observed this phenomenon, there is a lack of in-depth analysis and discussion of the factors contributing to this disparity. In this study, we used a Fairlie decomposition model to understand the factors that influence differences in depressive symptoms among older adults with hypertension in urban and rural areas. Participants were grouped based on their urban or rural status; demographic, social, personal lifestyle, and health indicators were used as covariates. This study aimed to analyze the differences in depressive symptoms between older patients with hypertension in urban and rural areas, decomposing these differences into contributions from different individual characteristic factors. The goal was to investigate the underlying causes of this inequality and provide theoretical evidence to promote health equity between urban and rural areas in China.

## Methods

### Sample Sizes and Data Sources

The data sources for this study were obtained from the 8th Chinese Longitudinal Health Longevity Survey (CLHLS), which was organized by Peking University in 2018 and made available to the public in 2020 [24]. This is the earliest social survey of the older population in China and has the largest number of older adult participants in the world. The project covered 23 provinces in China and included more than 110,000 participants between 1998 and 2018.

**Figure 1.**
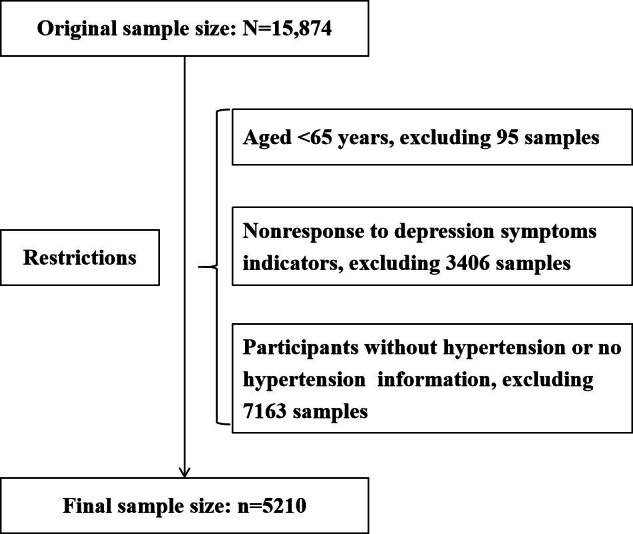
Flow chart of the study participants.

The sample size for the 8th CLHLS investigation in 2018 was 15,874 participants. Our study excluded participants who were younger than 65 years, did not respond to questions about depressive symptoms, did not respond to questions about hypertension, or had no hypertension. Finally, 5210 respondents were selected for statistical analysis. The exclusion process is illustrated in Figure 1.

### Measures

#### Depressive Symptoms

This study used the 10-item Center for Epidemiology Studies Depression Scale (CES-D) to evaluate depressive symptoms among the participants [25]. This scale has been validated by researchers, demonstrating sufficient reliability and validity for assessing depressive symptoms among older individuals in China [26]. Consistent with previous studies, we calculated the respondents’ scores for depressive symptoms on a 4-point scale, ranging from 0 (rarely or none of the time) to 3 (all of the time) [27]. The total score ranged from 0 to 30; participants with a total score of 10 or higher were classified as exhibiting depressive symptoms among older adults with hypertension [16]. In our study, the Cronbach α value of this scale was 0.73, and the Kaiser-Meyer-Olkin value of this scale was 0.85.

#### Hypertension

Respondents’ hypertension status was determined based on their responses to the question, “Do you suffer from hypertension as diagnosed by your doctor?” Those who answered affirmatively were classified as hypertensive older adults and were included in this study [28]; individuals who responded negatively or did not provide a response were excluded.

#### Grouping Variables

Participants were classiﬁed as rural or urban based on the nature of their domicile at the time of response.

### Covariates

To enhance the reliability of our findings, we controlled for several potentially influential factors derived from similar studies. Demographic characteristics, sociological factors, personal lifestyle factors, and health status indicators were considered, based on previous research on depressive symptoms. Demographic characteristics included age, gender, BMI, and years of education. Sociological factors included marital status, living status, and self-reported income status in local currency. Personal lifestyle factors included smoking status, alcohol consumption, exercise, and sleep duration. Health status indicators encompassed activities of daily living (ADL) dysfunction and comorbidities.

#### Demographic Characteristics

Age was categorized as <70, 70‐79, 80‐89, 90‐99, and ≥100 years. BMI was calculated by dividing weight (in kg) by the square of height (in meters) and categorized into 4 groups, as follows: <18.5, 18.5‐23.9, 24.0‐27.9, and ≥28.0 kg/m^2^ [16]. Years of education were classified as 0, 1‐6, and ≥7 years.

#### Sociological Factors

Marital status included being married and living with a spouse, being widowed, and other (including being married but not living with a spouse, divorced, and never married). Living status was classified as living with household members, living alone, or living in an institution. Self-reported income status was categorized as general, rich, or poor based on responses to the question, “How do you rate your economic status compared to other local residents?”

#### Personal Lifestyle Factors

Smoking, alcohol consumption, and exercise responses were categorized as yes or no based on participants’ responses to the questions, “Do you currently smoke?” “Do you drink alcohol at present?” and “Do you exercise regularly at present?” Sleep duration was classified as <4 hours, 4‐5.9 hours, 6‐7.9 hours, 8‐9.9 hours, and ≥10 hours, based on the answer to the question, “How many hours do you usually sleep?”

#### Health Status Indicators

We considered 8 categories of chronic diseases, as follows: diabetes, heart disease, stroke or cerebrovascular disease, chronic respiratory disease, gastric or duodenal ulcers, arthritis, dementia, and cholecystitis or cholelithiasis. The presence of one or more of these chronic diseases was used to define comorbidities among participants. Additionally, ADL is measured through 6 aspects in daily living ability, including bathing, dressing, bathroom use, indoor transferring, continence, and feeding. Each action is scored as follows: 0 for independent completion, 1 for completion with help, and 2 for total dependence on others. The sum of these scores, ranging from 0 to 12, categorizes ADL functioning as no dysfunction (0), mild dysfunction (1-6), or severe dysfunction (7-12) [29].

### Statistical Methods

Descriptive statistics were used to examine general information on demographic characteristics, sociological factors, personal lifestyle factors, and health status indicators. The *χ*^2^ test was used to investigate the distribution characteristics of depressive symptoms among the urban and rural older population with hypertension. The binary logistic regression model was used to explore the main determinants of depressive symptoms in rural and urban older residents with hypertension. Finally, the Fairlie model was applied to analyze the factors impacting and contributing to the disparities in depressive symptoms between the urban and rural older residents with hypertension. Furthermore, to ensure the robustness of our Fairlie model, we used a multiple imputation method to supplement the missing values of the covariates [30]; each missing value was supplemented 10 times. The Fairlie decomposition model is commonly used in economics to explain the underlying causes of a phenomenon [31]. The core concept is to decompose the overall difference into 2 parts—the explainable and the unexplainable part [32]. The explainable part is due to individual characteristics (eg, gender, race, and education level), whereas the unexplainable part is due to other unobserved factors (eg, market conditions and the environment) [33].

In this study, we used urban/rural status as the grouping variable and included 4 categories of covariate, as follows: demographic characteristics, sociological factors, personal lifestyle factors, and health status indicators. We calculated the regression coefficients of depressive symptoms in older patients with hypertension in urban and rural areas and used the decomposition formula to decompose the differences in depressive symptoms between urban and rural older populations with hypertension into the contributions of different individual characteristic factors [31]. The explainable part included statistically significant differences in the 4 categories of covariates, whereas the unexplainable part included the influence of unobserved factors on these differences.

The decomposition of the nonlinear equation $Y=F(X\hat{\beta })$ can be expressed as follows in equation (1):


(1) $${\overset{-}{Y}}^{a}-{\overset{-}{Y}}^{b}=\left[\sum_{i=1}^{N^{a}}\frac{F\left(X_{i}^{a}\beta^{a}\right)}{N^{a}}-\sum_{i=1}^{N^{b}}\frac{F\left(X_{i}^{b}\beta^{a}\right)}{N^{b}}\right]+\;\left[\sum_{i=1}^{N^{b}}\frac{F\left(X_{i}^{b}\beta^{a}\right)}{N^{b}}-\sum_{i=1}^{N^{b}}\frac{F\left(X_{i}^{b}\beta^{b}\right)}{N^{b}}\right]$$

${\overset{-}{Y}}^{a}$ and ${\overset{-}{Y}}^{b}$ represent the mean probabilities of depressive symptoms in the two groups; *F* is the cumulative distribution function of the logistic distribution; ${\overset{-}{Y}}^{a}-{\overset{-}{Y}}^{b}$ represents the total variation due to group differences; and $N^{a}$ and $N^{b}$ are the sample sizes of the two populations. The first term in parentheses in equation (1) represents the portion of the gap due to group differences in the observed characteristics and the portion attributable to differences in the estimated coefficients, whereas the second term represents the portion due to differences in Y levels.

### Ethical Considerations

This study is a secondary analysis of CLHLS, and the primary data collection of the survey were approved by the Ethics Committee of Peking University (IRB00001052-13074). The participants’ personal information and privacy were strictly protected by the CLHLS according to the rules set by the Peking University’s Biomedical Ethics Review Committee.

## Results

### Participants' Characteristics

The total sample size of this study was 5210, with 63.95% (n*=*3332) of the participants residing in rural areas. Table 1 presents the descriptive statistics of study participants. We observed that 12.84% (n*=*669) of the participants exhibited depressive symptoms, and the proportion of depressive symptoms in rural areas (14.05%) was significantly higher (*P*=.001) than in urban areas (10.70%). *χ*^2^ tests revealed significant differences (*P*<.05) in the distribution of 11 covariates, including gender, BMI, years of education, marital status, living arrangements, self-reported income status, smoking, exercise, sleep duration, ADL dysfunction, and comorbidities between older individuals with hypertension in urban and rural areas.

**Table 1.** Demographic characteristics of participants (N=5210).

Variables		Rural areas, n (%)	Urban areas, n (%)	*χ*^2^ test (*df*)	*P* value
**Depressive symptoms**				11.99 (1)	.001
	No	2864 (85.95)	1677 (89.30)		
	Yes	468 (14.05)	201 (10.70)		
**Age (years)**				6.98 (4)	.14
	<70	414 (12.42)	221 (11.77)		
	70‐79	1055 (31.66)	560 (29.82)		
	80‐89	1011 (30.34)	596 (31.74)		
	90‐99	597 (17.92)	376 (20.02)		
	≥100	255 (7.65)	125 (6.66)		
**Gender**				6.52 (1)	.01
	Male	1427 (42.83)	873 (46.49)		
	Female	1905 (57.17)	1005 (53.51)		
**BMI (kg/m** ^**2**^ **)**				66.37 (4)	<.001
	18.5‐23.9	1578 (47.36)	767 (40.84)		
	<18.5	353 (10.59)	115 (6.12)		
	24.0‐27.9	932 (27.97)	660 (35.14)		
	≥28.0	370 (11.10)	262 (13.95)		
	Missing	99 (2.97)	74 (3.94)		
**Years of education**				908.59 (3)	<.001
	0	1464 (43.94)	343 (18.26)		
	1‐6	1097 (32.92)	527 (28.06)		
	≥7	313 (9.39)	848 (45.15)		
	Missing	458 (13.75)	160 (8.52)		
**Marital status**				9.04 (3)	.01
	Married and living with spouse	1503 (45.11)	931 (49.57)		
	Widowed	1691 (50.75)	878 (46.75)		
	Other	100 (3.00)	59 (3.14)		
	Missing	38 (1.14)	10 (0.53)		
**Living status**				149.22 (3)	<.001
	Living with household members	2610 (78.33)	1434 (76.36)		
	Living alone	616 (18.49)	266 (14.16)		
	Living in an institution	60 (1.80)	165 (8.79)		
	Missing	46 (1.38)	13 (0.69)		
**Self-reported income status based on local currency**				175.69 (3)	<.001
	General	2334 (70.05)	1223 (65.12)		
	Rich	583 (17.50)	569 (30.30)		
	Poor	384 (11.52)	71 (3.78)		
	Missing	31 (0.93)	15 (0.80)		
**Smoking**				22.46 (2)	<.001
	No	2787 (83.64)	1656 (88.18)		
	Yes	512 (15.37)	200 (10.65)		
	Missing	33 (0.99)	22 (1.17)		
**Alcohol consumption**				2.86 (2)	.09
	No	2817 (84.54)	1623 (86.42)		
	Yes	462 (13.87)	230 (12.25)		
	Missing	53 (1.59)	25 (1.33)		
**Exercise**				195.12 (2)	<.001
	No	2277 (68.34)	924 (49.20)		
	Yes	1010 (30.31)	938 (49.95)		
	Missing	45 (1.35)	16 (0.85)		
**Sleep duration (hours)**				43.00 (5)	<.001
	<4.0	130 (3.90)	48 (2.56)		
	4.0‐5.9	559 (16.78)	319 (16.99)		
	6.0‐7.9	1097 (32.92)	732 (38.98)		
	8.0‐9.9	937 (28.12)	538 (28.65)		
	≥10.0	591 (17.74)	228 (12.14)		
	Missing	18 (0.54)	13 (0.69)		
**ADL**^**a**^ **dysfunction**				52.81 (3)	<.001
	No	2738 (82.17)	1404 (74.76)		
	Mild	380 (11.40)	326 (17.36)		
	Severe	82 (2.46)	81 (4.31)		
	Missing	132 (3.96)	67 (3.57)		
**Comorbidity**				201.81 (2)	<.001
	No	1430 (42.92)	461 (24.55)		
	Yes	1342 (40.28)	1110 (59.11)		
	Missing	560 (16.81)	307 (16.35)		

a ADL: activities of daily living.

### Variable Distribution by Depressive Symptoms Status

Table 2 shows the distribution of various variables according to depressive symptom status among older adults with hypertension in rural and urban areas. *χ*^2^ test results indicated that gender, marital status, smoking, exercise, and sleep duration exhibited distinct distribution patterns between hypertensive patients with and without depressive symptoms.

**Table 2.** Distribution of the variables in depressive symptoms and nondepressive symptoms among respondents (N=5210).

Variable		Nondepressive symptoms				Depressive symptoms			
		Rural areas, n (%)	Urban areas, n (%)	*χ*^2^ test (*df*)	*P* value	Rural areas, n (%)	Urban areas, n (%)	*χ*^2^ test (*df*)	*P* value
**Age (years)**				5.92 (4)	.21			7.85 (4)	.10
	<70	367 (12.81)	194 (11.57)			47 (10.04)	27 (13.43)		
	70‐79	920 (32.12)	505 (30.11)			135 (28.85)	55 (27.36)		
	80‐89	856 (29.89)	528 (31.48)			155 (33.12)	68 (33.83)		
	90‐99	514 (17.95)	334 (19.92)			83 (17.74)	42 (20.90)		
	≥100	207 (7.23)	116 (6.92)			48 (10.26)	9 (4.48)		
**Gender**				1.48 (1)	.22			11.47 (1)	.001
	Male	1294 (45.18)	789 (47.05)			133 (28.42)	84 (41.79)		
	Female	1570 (54.82)	888 (52.95)			335 (71.58)	117 (58.21)		
**BMI (kg/m^2^)**				55.46 (3)	<.001			8.07 (3)	.045
	18.5‐23.9	1354 (47.28)	688 (41.03)			224 (49.67)	79 (42.70)		
	<18.5	283 (9.88)	95 (5.66)			70 (15.52)	20 (10.81)		
	24.0‐27.9	824 (28.77)	601 (35.84)			108 (23.95)	59 (31.89)		
	≥28.0	321 (11.21)	235 (14.01)			49 (10.86)	27 (14.59)		
**Years of education**				728.52 (2)	<.001			196.08 (2)	<.001
	0	1198 (41.83)	312 (18.60)			266 (66.83)	31 (16.32)		
	1‐6	987 (34.46)	464 (27.67)			110 (27.64)	63 (33.16)		
	≥7	291 (10.16)	752 (44.84)			22 (5.53)	96 (50.53)		
**Marital status**				4.70 (2)	.10			6.97 (2)	.03
	Married and living with spouse	1341 (46.82)	840 (50.09)			162 (34.99)	91 (45.50)		
	Widowed	1406 (49.09)	773 (46.09)			285 (61.56)	105 (52.50)		
	Other	84 (2.93)	55 (3.28)			16 (3.46)	4 (2.00)		
**Living status**				108.47 (2)	<.001			48.89 (2)	<.001
	Living with household members	2280 (79.61)	1291 (76.98)			330 (71.90)	143 (71.14)		
	Living alone	495 (17.28)	237 (14.13)			121 (26.36)	29 (14.43)		
	Living in an institution	52 (1.82)	136 (8.11)			8 (1.74)	29 (14.43)		
**Self-reported income status based on local currency**				129.31 (2)	<.001			37.85 (2)	<.001
	General	2048 (71.51)	1084 (64.64)			286 (61.77)	139 (69.85)		
	Rich	542 (18.92)	529 (31.54)			41 (8.86)	40 (20.10)		
	Poor	248 (8.66)	51 (3.04)			136 (29.37)	20 (10.05)		
**Smoking**				21.97 (1)	<.001			1.62 (1)	.20
	No	2380 (83.10)	1474 (87.90)			407 (88.10)	182 (91.46)		
	Yes	457 (15.96)	183 (10.91)			55 (11.90)	17 (8.54)		
**Alcohol consumption**				6.50 (1)	.01			4.17 (1)	.04
	No	2389 (83.41)	1446 (86.23)			428 (93.25)	177 (88.50)		
	Yes	431 (15.05)	207 (12.34)			31 (6.75)	23 (11.50)		
**Exercise**				197.12 (1)	<.001			1.21 (1)	.27
	No	1926 (67.25)	778 (46.39)			351 (76.64)	146 (72.64)		
	Yes	903 (31.53)	883 (52.65)			107 (23.36)	55 (27.36)		
**Sleep duration (hours)**				40.71 (4)	<.001			5.71 (4)	.22
	<4.0	83 (2.90)	32 (1.91)			47 (10.09)	16 (7.96)		
	4.0‐5.9	401 (14.00)	250 (14.91)			158 (33.91)	69 (34.33)		
	6.0‐7.9	973 (33.97)	663 (39.53)			124 (26.61)	69 (34.33)		
	8.0‐9.9	849 (29.64)	510 (30.41)			88 (18.88)	28 (13.93)		
	≥10.0	542 (18.92)	209 (12.46)			49 (10.52)	19 (9.45)		
**ADL**^**a**^ **dysfunction**				46.54 (2)	<.001			14.45 (2)	.001
	No	2398 (83.73)	1285 (76.62)			340 (75.72)	119 (61.66)		
	Mild	297 (10.37)	275 (16.40)			83 (18.49)	51 (26.42)		
	Severe	56 (1.96)	58 (3.46)			26 (5.79)	23 (11.92)		
**Comorbidity**				176.14 (1)	<.001			30.81 (1)	<.001
	No	1272 (44.41)	428 (25.52)			158 (43.05)	33 (18.75)		
	Yes	1133 (39.56)	967 (57.66)			209 (56.95)	143 (81.25)		

a ADL: activities of daily living.

### Logistic Regression Analysis

Table 3 presents the binary logistic regression results for depressive symptoms in older adults with hypertension in rural and urban areas. The rural logistic model revealed that years of education (1‐6 years: odds ratio [OR] 0.68, 95% CI 1.10-1.21; ≥7 years: OR 0.47, 95% CI 0.24-0.94), alcohol consumption (yes: OR 0.52, 95% CI 0.29-0.93), exercise (yes: OR 0.78, 95% CI 0.56-1.08), and sleep duration (6.0‐7.9 hours: OR 0.29, 95% CI 0.17-0.52; 8.0‐9.9 hours: OR 0.24, 95% CI 0.13-0.43; ≥10.0 hours: OR 0.22, 95% CI 0.11-0.41) were protective factors against depressive symptoms in older adults with hypertension. In contrast, gender (female: OR 1.94, 95% CI 1.33-2.81), self-reported income status (poor: OR 3.07, 95% CI 2.16-4.37), and ADL dysfunction (mild: OR 1.69, 95% CI 1.11-2.58; severe: OR 3.03, 95% CI 1.46-6.32) were identified as risk factors. In the urban logistic model, age (90‐99 years: OR 0.37, 95% CI 0.16-0.81; ≥100 years: OR 0.19, 95% CI 0.06-0.66), exercise (yes: OR 0.33, 95% CI 0.22-0.51), and sleep duration (6.0-7.9 hours: OR 0.27, 95% CI 0.10-0.71; 8.0‐9.9 hours: OR 0.16, 95% CI 0.06-0.44; ≥10.0 hours: OR 0.18, 95% CI 0.06-0.57) were protective factors against depressive symptoms in older adults with hypertension. Conversely, years of education (1‐6 years: OR 1.91, 95% CI 1.05-3.49), self-reported income status (poor: OR 2.94, 95% CI 1.43-6.08), and ADL dysfunction (mild: OR 2.38, 95% CI 1.39-4.06; severe: OR 3.26, 95% CI 1.21-8.76) were identified as risk factors.

**Table 3.** Binary logistic regression analysis of depressive symptoms in older patients with hypertension residing in rural and urban areas (N=5210).

Variables		Rural areas		Urban areas	
		OR^a^ (95% CI)	*P* value	OR (95% CI)	*P* value
**Age (years)**					
	<70	Reference	—^b^	Reference	—
	70‐79	1.10 (0.69-1.76)	.70	0.78 (0.43-1.43)	.42
	80‐89	0.84 (0.50-1.39)	.49	0.65 (0.34-1.25)	.19
	90‐99	0.55 (0.30-1.01)	.05	0.37 (0.16-0.81)	.01
	≥100	0.69 (0.33-1.42)	.32	0.19 (0.06-0.66)	.01
**Gender**					
	Male	Reference	—	Reference	—
	Female	1.94 (1.33-2.81)	.001	0.83 (0.53-1.31)	.43
**BMI (kg/m^2^)**					
	18.5‐23.9	Reference	—	Reference	—
	<18.5	1.15 (0.74-1.81)	.54	1.19 (0.57-2.47)	.65
	24.0‐27.9	0.84 (0.60-1.18)	.32	0.95 (0.62-1.47)	.83
	≥28.0	0.83 (0.52-1.33)	.44	0.79 (0.43-1.47)	.46
**Years of education**					
	0	Reference	—	Reference	—
	1‐6	0.68 (0.49-0.95)	.02	1.91 (1.05-3.49)	.04
	≥7	0.47 (0.24-0.94)	.03	1.71 (0.92-3.17)	.09
**Marital status**					
	Married and living with spouse	Reference	—	Reference	—
	Widowed	1.21 (0.84-,1.74)	.32	1.12 (0.66-1.91)	.68
	Other	1.38 (0.57-3.37)	.48	0.22 (0.03-1.81)	.16
**Living status**					
	Living with household members	Reference	—	Reference	—
	Living alone	1.41 (0.97-2.04)	.07	1.08 (0.58-1.99)	.81
	Living in an institution	0.39 (0.10-1.48)	.17	1.27 (0.61-2.62)	.53
**Self-reported income status based on local currency**					
	General	Reference	—	Reference	—
	Rich	0.64 (0.40-1.00)	.05	0.72 (0.45-1.15)	.17
	Poor	3.07 (2.16-4.37)	<.001	2.94 (1.43-6.08)	.004
**Smoking**					
	No	Reference		Reference	—
	Yes	1.26 (0.79-2.02)	.34	0.50 (0.24-1.05)	.07
**Alcohol consumption**					
	No	Reference	—	Reference	—
	Yes	0.52 (0.29-0.93)	.03	1.33 (0.71-2.50)	.37
**Exercise**					
	No	Reference	—	Reference	—
	Yes	0.78 (0.56-1.08)	.13	0.33 (0.22-0.51)	<.001
**Sleep duration (hours)**					
	<4.0	Reference	—	Reference	—
	4.0‐5.9	0.83 (0.47-1.46)	.51	0.84 (0.32-2.25)	.73
	6.0‐7.9	0.29 (0.17-0.52)	<.001	0.266 (0.10-0.74)	.01
	8.0‐9.9	0.24 (0.13-0.43)	<.001	0.16 (0.06-0.44)	<.001
	≥10.0	0.22 (0.11-0.41)	<.001	0.18 (0.06-0.57)	.003
**ADL**^**c**^ **dysfunction**					
	No	Reference	—	Reference	—
	Mild	1.69 (1.11-2.58)	.02	2.38 (1.39-4.07)	.002
	Severe	3.03 (1.46-6.32)	.003	3.26 (1.21-8.76)	.02
**Comorbidity**					
	No	Reference	—	Reference	—
	Yes	1.26 (0.95-1.68)	.12	1.32 (0.83-2.10)	.24

a OR: odds ratio.

b Not applicable.

c ADL: activities of daily living.

Thus, the divergent results indicate that certain protective and risk factors for depressive symptoms were not consistent between older adults with hypertension in rural and urban areas, particularly in terms of age, gender, years of education, and alcohol consumption.

### Fairlie Decomposition Results

Table 4 displays the outcomes of the Fairlie decomposition analysis for the disparity in depressive symptoms among older adults with hypertension, categorized by rural versus urban settings. The Fairlie decomposition analysis revealed that 91.61% of the disparity in depressive symptoms between older adults with hypertension in urban and rural areas was attributable to the covariates considered in this study. To ensure the robustness of our Fairlie model, we also constructed a multiple imputation model for supplementary analysis (Table S1 in Multimedia Appendix 1), and the results of Table S1 in Multimedia Appendix 1 show the same significance of the variables, indicating that our Fairlie model results were sufficiently robust. However, these findings demonstrated that years of education (contribution 63.07%), self-reported income status (contribution 13.24%), exercise (contribution 45.65%), sleep duration (contribution 20.77%), ADL dysfunction (contribution −9.64%), and comorbidity (contribution −22.94%) were statistically significant in explaining the observed differences.

**Table 4.** The Fairlie decomposition of depressive symptoms disparity between older patients with hypertension residing in rural areas and those residing in urban areas.

Contribution to difference		*P* value	β	Contribution (%; 95%CI)	
Age		.07	–0.0016597	–9.84 (–0.0034193 to 0.0000999)	
Gender		.27	0.0003710	2.20 (–0.0002921 to 0.0010341)	
BMI		.16	0.0008204	4.87 (–0.0003261 to 0.0019668)	
Years of education		.04	0.0107388	63.70 (0.0005549 to 0.0209226)	
Marital status		.65	0.0001120	0.66 (–0.0003716 to 0.0005956)	
Living status		.21	–0.0019643	–11.65 (–0.0050002 to 0.0010715)	
Self-reported income status based on local currency		.001	0.0022317	13.24 (0.0008965 to 0.0035669)	
Smoking		.78	–0.0002141	–1.27 (–0.0017374 to 0.0013092)	
Alcohol consumption		.10	–0.0009192	–5.45 (–0.0020117 to 0.0001733)	
Exercise		<.001	0.0076954	45.65 (0.0044708 to 0.0109200)	
Sleep duration		.01	0.0035009	20.77 (0.0010611 to 0.0059406)	
ADL^a^ dysfunction		.01	–0.0016250	–9.64 (–0.0028419 to 0.0004080)	
Comorbidity		.03	–0.0038677	–22.94 (–0.0074016 to 0.0003338)	

a ADL: activities of daily living.

## Discussion

### Principal Findings

To the best of our knowledge, this is the first study to investigate the disparities in depressive symptoms among older Chinese patients with hypertension living in rural and urban areas. This study included demographic characteristics, sociological factors, personal lifestyle factors, and health status indicators as influencing factors and built a decomposition model with an explanatory power of 91.61%, providing new empirical evidence for the prevention and treatment of depressive symptoms in older patients with hypertension in China. Specifically, years of education (63.07%), self-reported income status (13.24%), exercise (45.65%), sleep duration (20.77%), ADL dysfunction (−9.64%), and comorbidity (−22.94%) were statistically significant in explaining the observed differences.

Our study showed that older patients with hypertension in rural areas were more likely to have depressive symptoms than those in urban areas, which is consistent with previous research [15]. In this survey, 12.84% of the participants exhibited depressive symptoms, which was higher than the reported proportion of depressive symptoms among the general older population (11.72%) in a previous study [16]. Moreover, older patients with hypertension were more susceptible to depressive symptoms than the general older population (*P*=.04). Interestingly, our study observed a notable discrepancy, indicating that older adults with hypertension residing in rural areas exhibited significantly higher rates of depression (14.05%) than the general older adult population reported in previous studies (12.41%) [16]. However, this increased percentage of depression was not observed in older adults with hypertension living in urban settings. This result is consistent with existing research in countries such as Norway [34], India [35], and Mexico [36], which report that residents in rural areas are more prone to depressive symptoms. Based on this evidence, our study suggests that family physicians working in rural areas should provide appropriate psychological counseling for older hypertensive patients during their treatment, while public health personnel in rural areas should pay attention to the mental health education of hypertensive patients to help prevent the occurrence of depressive symptoms.

In addition, our logistic model results showed that the factors influencing depressive symptoms among patients with hypertension in rural and urban areas in China had both similarities and differences. The similarities mainly included exercise habits and sleep duration of more than 6 hours, which reduced their risk of depressive symptoms, while poverty and ADL dysfunction increased their risk of depressive symptoms. The differences mainly included education level (older patients with hypertension who received 1‐6 years of education in rural areas had a lower risk of depression symptoms, while those who received 1‐6 years of education in urban areas had an increased risk), alcohol consumption (a protective factor in rural areas but not significant in urban areas), age (risk decreased in urban areas for those older than 90 years but not in rural areas), and gender (increased risk among female individuals in rural areas but not significant in urban areas). Our findings are similar to those of several international scholars and confirm that economic status, ADL dysfunction, exercise, and education are the main factors influencing depressive symptoms in patients with hypertension [9,35,37]. At the same time, our findings suggest that China needs to implement different programs for hypertensive patients in rural and urban areas, taking into account the different risk factors for depressive symptoms in policy formulation.

Most importantly, our study identified the main reasons for the differences in depressive symptoms among older patients with hypertension in rural and urban areas in China. The results showed that years of education (63.07%), self-reported income status (13.24%), exercise (45.65%), sleep duration (20.77%), ADL dysfunction (−9.64%), and comorbidity (−22.94%) were the main factors influencing this difference. Fortunately, these factors are all intervenable, especially the two main factors of years of education and exercise [37,38], which have been reported in previous studies to be significantly higher in older people in urban areas compared to those in rural areas because of better economic conditions and living environments in urban areas [39,40]. Older people in urban areas mostly live in communities that provide basic fitness facilities and equipment to help maintain their physical health, whereas rural areas in China lack such exercise venues and equipment [41]. The Chinese government needs to strengthen health education for older adults in rural areas and provide fitness equipment for them to exercise while paying attention to groups with less sleep time and poorer economic conditions [42,43]. Whether in urban or rural areas, attention needs to be paid to older people with ADL dysfunction and comorbidities [44]. Through various means and channels, governments can reduce the risk of depressive symptoms among older people in China’s urban and rural areas to promote the psychological health equity of older people in China.

In conclusion, the Chinese government should prioritize health education in rural areas, provide information resources, and conduct outreach programs to emphasize the importance of a healthy lifestyle, balanced nutrition, and regular exercise. Furthermore, the government should address inadequate sleep among economically disadvantaged groups by offering financial aid, scholarships, and discounted health care services to improve living conditions and access to health care. These measures will effectively enhance the overall well-being and mental health of the rural population, especially vulnerable groups that face greater challenges in accessing resources and support.

### Limitations

Our study had some limitations. First, we surveyed only a portion of hypertensive patients in China, which has a large population; therefore, our study only reflects the situation of the participants included in this study. Second, we used the 10-item CES-D, which is measured subjectively and may have resulted in measurement bias. Third, there are many factors affecting depression symptoms, and we included only some of them. Our team will consider these limitations and continue to improve future studies to make our research more fully supported by evidence.

### Conclusions

To the best of our knowledge, this study is the first to investigate the disparities in depressive symptoms among older Chinese patients with hypertension living in rural and urban areas. The results of our study suggest that older hypertensive patients in rural areas are more susceptible to depression symptoms than those in urban areas; the factors influencing depressive symptoms among hypertensive patients in rural and urban areas in China had similarities in terms of exercise, sleep duration, self-reported income status, and ADL dysfunction, as well as differences in terms of age, gender, years of education, and alcohol consumption. Our study also identified the main reasons for the difference in depression symptoms among older hypertensive patients in rural and urban areas in China, which provides evidence for policy formulation to promote psychological health equity among older people in China.

## Supplementary material

10.2196/52536 Multimedia Appendix 1 The results of Fairlie decomposition with the multiple imputation method.

## Abbreviations

CES-D: Center for Epidemiological Survey Depression Scale
CLHLS: Chinese Longitudinal Healthy Longevity Survey
OR: odds ratio
