# Supplementary material for: Decomposition Analysis of Depressive Symptom Differences Among Older Adults With Hypertension Between Urban and Rural Areas: Cross-Sectional Study
Source: JMIR Public Health Surveill. 2024 Aug 1;10:e52536. doi: 10.2196/52536 (PMC11303882; doi:10.2196/52536)
Supplement: Multimedia Appendix 1 [file publichealth-v10-e52536-s001.docx]

**Table S1. The results of Fairlie decomposition with the multiple imputation method**

| Contribution to difference | *P* | *β* | (95%CI) |
| --- | --- | --- | --- |
| Age | 0.293 | -0.0003899 | (-0.0011169,0.0003371) |
| Gender | 0.292 | -0.0003520 | (-0.0010072,0.0003032) |
| BMI | 0.104 | 0.0011691 | (-0.0002386,0.0025768) |
| Years of school | 0.009 | 0.0111283 | (0.0027519,0.0195047) |
| Marital status | 0.290 | -0.0002634 | (-0.0007511,0.0002243) |
| Living status | 0.112 | -0.0024580 | (-0.0054930,0.0005771) |
| Self-report income status in local | <0.001 | 0.0029881 | (0.0017091,0.0042670) |
| Smoking | 0.772 | 0.0002104 | (-0.0012114,0.0016322) |
| Drinking | 0.069 | -0.0007586 | (-0.0015763,0.0000592) |
| Exercise | <0.001 | 0.0071624 | (0.0044965,0.0098284) |
| Sleep time | 0.002 | 0.0028963 | (0.0010417,0.0047509) |
| ADL dysfunction | <0.001 | -0.0035146 | (-0.0048941,-0.0021351) |
| Comorbidity | 0.001 | -0.0050573 | (-0.0081434,-0.0019713) |
